# Modulation of IL-1β reprogrammes the tumor microenvironment to interrupt oral carcinogenesis

**DOI:** 10.1038/srep20208

**Published:** 2016-02-01

**Authors:** Tong Wu, Yun Hong, Lihua Jia, Jie Wu, Juan Xia, Juan Wang, Qinchao Hu, Bin Cheng

**Affiliations:** 1 Department of Oral Medicine, Hospital of Stomatology, Sun Yat-sen University, Guangzhou, China; 2 Guangdong Provincial Key Laboratory of Stomatology, Guanghua School of Stomatology, Sun Yat-sen University, Guangzhou, China

## Abstract

Head and neck squamous cell carcinoma (HNSCC) development is a multistage process includes the normal, dysplasia and squamous cell carcinoma (SCC) stages. Recently, increasing evidence has suggested that the tumor microenvironment (TME) is an integral part of malignant transformation. Exploring certain key node genes in TME for future intervention in dysplasia to interrupt oral carcinogenesis was the primary goal of this research. To achieve this goal, systems biology approaches were first applied to the epithelia and fibroblasts collected at sequential stages in a 4-nitroquinoline-1-oxide (4NQO) - induced rat oral carcinogenesis model. Through bioinformatics network construction, IL-1β was identified as one of the key node genes in TME during carcinogenesis. Immunohistochemical staining of human and rat samples demonstrated that IL-1β expression patterns were parallel to the stages of malignant transformation. Silencing IL-1β with lentivirus-delivered shRNA significantly inhibited oral squamous cell carcinoma cell growth both *in vivo* and *in vitro*. Based on these findings, we hypothesized that IL-1β may be a chemoprevention target in TME during oral carcinogenesis. Therefore, we targeted IL-1 in the TME by oral mucosal injection of an IL-1 receptor antagonist in 4NQO rats. The results demonstrated that targeting IL-1 could interrupt oral carcinogenesis by reprogramming the TME.

Head and neck squamous cell carcinoma (HNSCC) is the sixth most common cancer in the world, accounting for 650,000 new cases and 350,000 deaths each year^1^. Despite several advances in therapeutic regimens, HNSCC remains characterized by a poor prognosis and low survival rate^2^.

HNSCC development is a multistage process that includes the normal, dysplasia and squamous cell carcinoma (SCC) stages. Considering the easy accessibility of oral lesions, the dysplasia stage provides a prime opportunity to interrupt the carcinogenesis process. Recently, increasing evidence has suggested that the tumor microenvironment (TME) is an integral and inseparable part of malignant transformation^3^,^4^,^5^. The TME is formed by stromal cells, infiltrating immune cells, and extra-cellular components around tumor cells that can promote carcinogenesis. Oral carcinogenesis is a complex and multifaceted process; the acquisition of malignant features within a favorable TME occurs along with behavioral changes of epithelia and fibroblasts that typically involve a broad range of dynamic variations. Strategies aimed at interfering with the cross-talk between tumor cells and their cellular partners in the TME have been considered for the development of a novel cancer therapy^5^,^6^.

Because all the components of the TME work as a complex network, the identification of key node genes in the TME is necessary for future intervention at the dysplasia stage to interrupt oral carcinogenesis for clinical purposes. To achieve this goal, a reliable animal model is a prerequisite. The 4-nitroquinoline-1-oxide (4NQO) rat model is one of the most extensively studied animal systems because of its close similarity to human malignant transformation at the histological and molecular levels^7^. This animal model facilitates our ability to observe, discover and confirm additional details about malignant transformation and will greatly contribute to future therapeutic applications.

Because of the complexity of the TME, high-throughput technology and systems biology approaches were applied in the 4NQO-induced rat oral carcinogenesis model. Through bioinformatics network construction and subsequent verification, interleukin 1 beta (IL-1β) was identified as one of the key node genes during carcinogenesis. Based on this finding, we hypothesized that IL-1β may be chemoprevention target in the TME during oral carcinogenesis. Therefore, an IL-1 receptor antagonist (IL-1Ra), which inhibits either IL-1α or IL-1β by competitively blocking IL-1 receptor I, was used to interrupt 4NQO-induced carcinogenesis^8^. The results demonstrated that IL-1Ra intervention could interrupt tumor progression by reprogramming the TME.

## Results

### IL-1β acts as a key node gene in the TME during oral carcinogenesis

**Figure 1:**
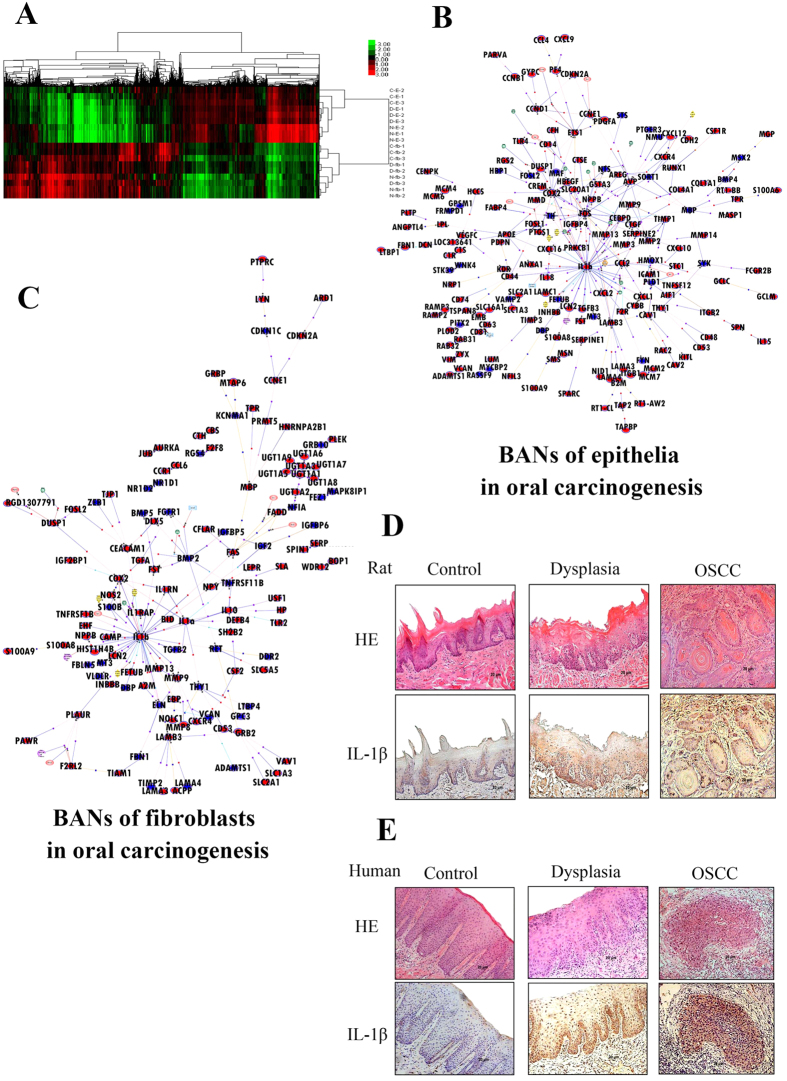
IL-1β acts as a key node gene in the microenvironment during oral carcinogenesis. (**A**) Epithelia and fibroblasts from the sequential stages of neoplasia including the normal, dysplasia and squamous cell carcinoma (SCC) stages (three rat epithelia/fibroblasts per sample, and three samples per group) were collected for microarray and hierarchical clustering analysis (N-E-1,2,3 from normal, D-E-1,2,3 from dysplasia and C-E-1,2,3 from carcinoma epithelia; N-fb-1,2,3 from normal, D-fb-1,2,3 from dysplasia and C-fb-1,2,3 from carcinoma fibroblasts). (**B,C**) Biological associated networks (BANs) were constructed by GeneSpringGX software according to the microarray data of epithelia (**B**) and fibroblast (**C**) from normal to SCC. Upregulated node genes are marked in red and downregulated node genes are marked in blue. (**D,E**) Representative images of IL-1β immunohistochemical staining in normal, dysplasia and OSCC epithelia from 4NQO-induced oral carcinogenesis rat model (**D**) and human oral cavity (**E**) (magnification 200×).

To explore key node genes in the TME during oral carcinogenesis, epithelia and fibroblasts from the sequential stages of neoplasia, namely the normal, dysplasia and SCC stages (three rat epithelia/fibroblasts per sample, three samples per group), were collected for microarray analysis (GSE42780,GSE42780). In the oral epithelia, 839 probe sets were found to be differentially expressed from the normal to the dysplasia state (449 up-regulated and 390 down-regulated) and 525 changes occurred from the dysplasia to the SCC state (440 up-regulated and 125 down-regulated). In submucosal fibroblasts, 563 probe sets were identified as differentially expressed from the normal to the dysplasia state (253 up-regulated and 313 down-regulated) and 714 changes occurred (642 up-regulated and 72 down-regulated) from the dysplasia to the carcinoma state. These observations were confirmed by RT-PCR (Supplementary Table 1, Supplementary Fig. 1). Clustering analysis showed that epithelial samples from the same group (normal, dysplasia and SCC) clustered together, while one fibroblasts sample from the dysplasia group clustered with the normal group (Fig. 1A).

To provide a functional context for the differentially expressed genes during carcinogenesis, GeneSpringGX software v11.0 (Agilent Technologies) was employed to construct biological association networks (BANs). In epithelia from the normal to SCC groups, 192 network eligible genes and 387 interactions were involved in BANs (Fig. 1B). In fibroblasts 138 network eligible genes and 235 interactions were detected (Fig. 1C). The BANs construction identified several highly interconnected nodes, such as IL-1β and cyclooxygenase-2 (COX2). Interestingly, IL-1β was one of the mostly highly interconnected nodes in both the epithelial and fibroblast networks, and it interconnected with a subnetwork that included TGFβ, COX2, CXCL2, MMP9, MMP13, and CCL2.

To detect IL-1β expression during carcinogenesis in the rat oral mucosa, immunohistochemical staining was employed. The number of IL-1β-positive cells gradually increased in accordance with malignant transformation in the oral epithelia. The frequency of IL-1β-positive cells in normal (36.8% ± 6.1%) and dysplasia (80.4% ± 6.3%) samples was significantly lower than that in SCC samples (96.5% ± 3.1%) (*P* < 0.05) (Fig. 1D).

To test whether the IL-1β expression pattern in humans is similar to that in 4NQO-induced carcinogenesis, oral mucosa samples from healthy, leukoplakia, and SCC subjects were collected for immunohistochemical analysis. As shown in Fig. 1E, in normal oral mucosa, only weak IL-1β staining was detected (the frequency of IL-1β positive cells was 28.8% ± 9.1%). In the leukoplakia sample, IL-1β staining was modestly higher (the frequency of IL-1β-positive cells was 78.4% ± 7.3%). However, staining of IL-1β was particularly prominent in the abundant epithelial cytoplasm in SCC samples (the frequency of positive cells was 91.5% ± 2.9%), where it was significantly higher than in normal and leukoplakia samples (*P* < 0.05). In sum, the IL-1β expression pattern was closely associated with malignant transformation and was one of the key node genes in the microenvironment during carcinogenesis.

### Silencing IL-1β inhibits oral squamous cell carcinoma cell growth *in vitro* and *in vivo*

**Figure 2:**
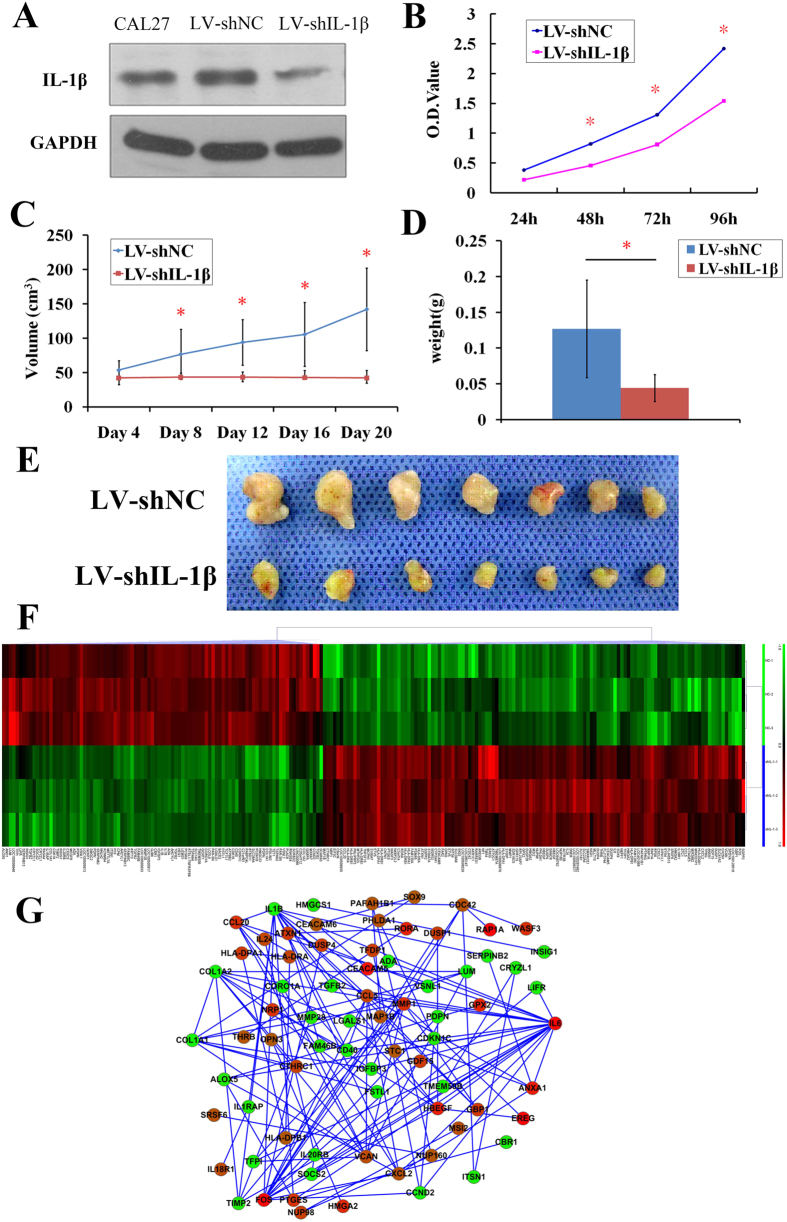
Silencing IL-1β inhibits OSCC growth *in vitro* and *in vivo*. (**A**) IL-1β protein expression levels in Cal27 cells after transfected with IL-1β shRNA (LV-shIL-1β) or LV-shNC. (**B**) After transfected with LV-shIL-1β or LV-shNC, Cal27 cells cell viability was measured every 24 h. (*P < 0.05 vs. cells transfected with LV-shNC) (**C,D**) After transfected with LV-shIL-1β or LV-shNC, Cal27 were subcutaneously injected into the flank region of nude mice. Tumor volumes were measured once every 4 days. 20 days later the mice were sacrificed and the tumors were removed and weighed. Tumor volumes (**C**) and weights (**D**) are presented as the mean ± SD. (*P < 0.05 vs. control). (**E**) Representative images of xenograft tumors obtained after the 20th day from Cal27 inoculation. (**F**) Microarray analysis of Cal27 cells transfected with LV-shIL-1β or LV-shNC. ShIL-1-1, 2, 3 from LV-shIL-1β group, NC-1, 2, 3 from LV-shNC group. (**G**) A total of 125 significantly changed central node genes were selected from above Cal27 microarray data to construct a multicenter interaction network. Upregulated node genes are marked in red and downregulated node genes are marked in blue.

To determine the role of IL-1β in regulating the growth of oral squamous cell carcinoma cells, lentivirus-mediated IL-1β shRNAs (LV-shIL-1β) and scramble shRNAs (LV-shNC) were transfected into Cal27 cells. IL-1β expression in LV-shIL-1β Cal27 cells was significantly decreased compared with that in control cells with LV-shNC (Fig. 2A). After LV-shIL-1β transfection, the growth of Cal27 cells after 48 h was inhibited compared with the growth of cells transfected with LV-shNC (*P* < 0.05) (Fig. 2B).

The effect of IL-1β shRNAs on tumor growth *in vivo* was evaluated using cancer xenograft volume changes. The tumor volume of each group was scored every 4 days, and the data are presented as mean values ± SD. LV-shIL-1β significantly inhibited tumor growth after day 8. Tumors from mice with LV- shIL-1β were significantly smaller in volume and weight than those in mice with LV-shNC (*P* < 0.05) (Fig. 2C,D,E).

To explore the variations in key node genes expression after LV-shIL-1β transfection, Cal27 cells transfected with LV-shIL-1β or LV-shNC were collected for microarray analysis. Compared with the LV-shNC group, 245 probe sets were found to be differentially expressed (>1.5 fold changed, *P* < 0.05) in the LV-shIL-1β group (140 up-regulated and 105 down-regulated) (GSE70301). These observations were confirmed by RT-PCR (Supplementary Table 1, Supplementary Fig. 2). Samples from the same experimental group clustered together, and the gene expression pattern was different after LV-shIL-1β transfection (Fig. 2F). The differentially expressed genes are involved in many Kyoto Encyclopedia of Genes and Genomes (KEGG) pathways associated with cancer such as the cytokine-cytokine receptor interaction, the MAPK signaling pathway and the Jak-STAT signaling pathway.

To obtain additional details about the kinetics and interactions among these differentially expressed genes, a multicenter interaction network was constructed. Of the 245 differentially expressed genes, the products of 151 genes formed a complex multicenter interaction network after STRING screening. After screening with Cytoscape, 125 central nodes (94 upregulated, 31 downregulated) were selected from the network (Fig. 2G). In this network some node genes that have been identified *in vivo* in the 4NQO rat model, such as TGFβ, were also identified as downstream genes of IL-1β. IL-1β may associate with these genes to generate a programmed, tumor favorable microenvironment during oral carcinogenesis.

### Targeting IL-1 by IL-1Ra interrupts oral carcinogenesis by modulation the TME

**Figure 3:**
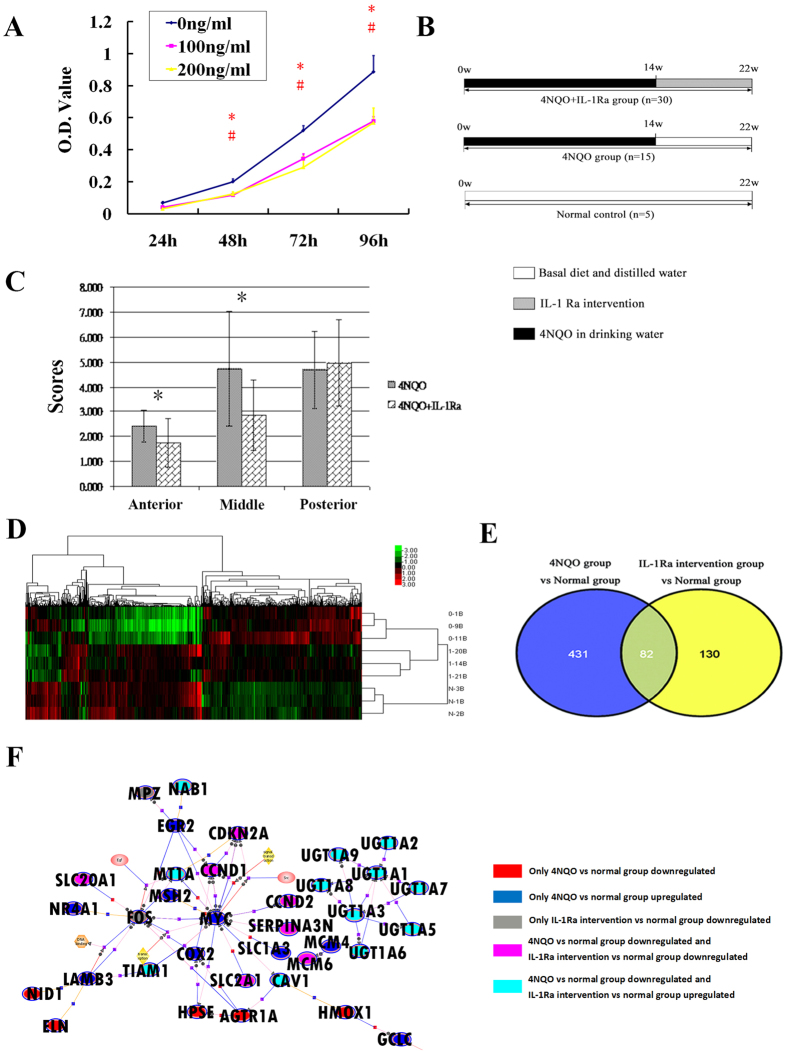
Targeting IL-1 by IL-1Ra interrupts oral carcinogenesis by modulating the microenvironment. (**A**) Cal27 cells were treated with the indicated concentrations of IL-1Ra. Cell viability was then measured at 24 h,48 h,76 h and 96 h. (*P < 0.05, 100 ng/ml IL-1Ra vs control; ^#^*P* < 0.05, 200 ng/ml IL-1Ra vs control). (**B**) Schematic drawing of IL-1Ra intervention during 4NQO induced rat tongue carcinogenesis. Rats were exposed to 4NQO for 14 weeks and then randomly distributed into 4NQO control and IL-1Ra intervention group. IL-1Ra intervention group (n = 30) received a tongue submucosal injection of IL-1Ra, while the 4NQO group (n = 15) received an equal volume of the diluent (sterile saline) weekly from week 14. At the end of week 22, the rats were sacrificed and tongues were dissected for histological analysis and microarray analysis. (**C**) The histological comparison between the IL-1Ra intervention and 4NQO groups (*P < 0.05). (**D**) Hierarchical clustering analysis after microarray experiment. 0–1B, 0–9B, and 0–11B from the 4NQO group; 1–20B, 1–14B and 1–21B from the IL-1Ra intervention group; and N-1,2,3B from the normal group. (**E**) Comparison of differentially expressed genes in the 4NQO group and IL-1Ra intervention group by Venn diagram. 431 genes were differentially expressed in the 4NQO group but not changed in IL-1Ra intervention group. (**F**) With the 431 genes, biological associated networks (BANs) were constructed by GeneSpringGX software.

After confirming the significant role of IL-1β in oral carcinogenesis both *in vitro* and *in vivo*, we wondered whether targeting IL-1 in the TME could inhibit malignant transformation. IL-1 receptor antagonists are some of the most commonly used therapeutic agents to target IL-1 by blocking IL-1 receptor I, which was expressed on oral mucosa (Supplementary Fig. 3 and Supplementary Fig. 4). First, we confirmed the effect of IL-1Ra *in vitro*. IL-1Ra significantly inhibited Cal27 cell growth after 48 h (Fig. 3A). Second, IL-1Ra was injected submucosally into the tongue during 4NQO-induced carcinogenesis to hinder malignant transformation (Fig. 3B). After IL-1Ra intervention, the malignant transformation process was interrupted. A histological comparison between the 4NQO and IL-1Ra intervention groups showed that the mean scores of the anterior (2.40 ± 0.63 vs 1.75 ± 0.97) and middle (4.73 ± 2.71 vs 2.86 ± 1.43) thirds of the rat tongue were significantly lower in the IL-1Ra intervention group (*P* < 0.05) (Fig. 3C). Thus, local injection of IL-1Ra can to some extent interrupt malignant transformation in the oral mucosa.

To explore the involvement of IL-1Ra intervention in modulating the microenvironment in oral carcinogenesis in more detail, nine rat tongue tissues (three samples per group) were randomly selected for microarray analysis. After IL-1Ra intervention, 290 differentially expressed genes were identified comparing with 4NQO group and 212 differentially expressed genes were identified compared with the normal group (GSE42855). These observations were confirmed by RT-PCR (Supplementary Table 1, Supplementary Fig. 5). To explore the differences in mRNA abundance among the normal, 4NQO and IL-1Ra intervention groups, hierarchical clustering was performed on the microarray data. The results showed that samples from the same experimental group clustered together; after IL-1Ra administration, the gene expression pattern was more similar to that of the normal group, indicating that the microenvironment was changed after IL-1Ra injection (Fig. 3D). The dynamic expression patterns of these genes may reveal details about the inhibition by IL-1Ra during malignant transformation. Therefore, the genes were classified according to their expression patterns during carcinogenesis. As the Venn diagram Fig. 3E shows, 431 genes that were differentially expressed in the 4NQO group but that were not differentially expressed during IL-1Ra intervention are of interest. The modulation of these genes may be the driving force behind IL-1Ra induced modulation of the TME interrupting malignant transformation. To facilitate a biological interpretation for these genes, we performed KEGG analysis. Notably, these genes were involved in many KEGG pathways that are associated with cancer, such as endometrial cancer, prostate cancer, colorectal cancer, non-small cell lung cancer, and the mTOR signaling pathway. Furthermore, to obtain additional details about the kinetics and interactions among these genes, BANs were constructed (Fig. 3F). Interestingly, the oncogene Myc and the inflammation-associated gene COX2 were the most highly interconnected nodes that were up-regulated in 4NQO samples but downregulated significantly after IL-1Ra injection. These results imply that IL-1Ra local injection can regulate the expression of key node genes in the TME, thereby leading to the interruption of malignant transformation in the oral mucosa.

## Discussion

It is now well recognized that the TME has played a significant role in cancer progression, enabling primary, invasive, and then metastatic growth^3^,^4^,^5^. The TME refers to the dynamic cellular and extra-cellular components that surround tumor cells at each stage of carcinogenesis; it consist of a complex network of fibroblasts, endothelial cells, blood vessels and immune cells, along with secreted factors such as cytokines, growth factors and numerous extracellular matrix (ECM) components^9^,^10^.

Oral carcinogenesis is a complex and multifaceted process, and the acquisition of malignant features occurs within a favorable TME, in which the behavioral changes of epithelia and fibroblasts typically involve a broad range of dynamic variations. The 4NQO rat cancer model is one of the most extensively studied animal systems for its close similarity to human malignant transformation at the histological and molecular levels^7^. To obtain additional details about the gene interactions and to explore key node genes in the TME during oral carcinogenesis, BANs were constructed by compared samples from the normal to SCC stages. One of the most exciting findings in this study is that, by using-high throughput bioinformatics analysis and visualized network construction, IL-1β was identified as one of the mostly highly interconnected nodes in both epithelial and fibroblast networks. These networks are interconnected with a subnetwork of genes that are associated with cancer progression, such as COX2, TGFβ, CCL2, CXCL2, MMP9, and MMP13. Similar bioinformatics research strategies have been applied to cervical cancer to successfully explore the key node genes that initiate carcinogenesis^11^.

IL-1β, a typical cancer-inflammation linked cytokine, is reportedly up-regulated in several types of tumors, including breast, colon, lung and esophageal cancers, and it was also shown to be increased in saliva and tissue from oral squamous cell carcinoma (OSCC) patients^8^,^12^,^13^,^14^,^15^,^16^,^17^,^18^. In our 4NQO rat model and in human oral samples, the expression patterns of IL-1β were found to be closely associated with malignant transformation. Together with our high-throughput results, we concluded that IL-1β is one of the key node genes behind the generation of a pro-tumorigenic microenvironment that drives oral carcinogenesis. To further validate our hypothesis, we confirmed the effect of IL-1β on HNSCC cells *in vitro*. Our results demonstrated that IL-1β knockdown significantly inhibited OSCC cell growth *in vitro* and *in vivo* and activated downstream genes, such as TGFβ, that were also identified in the 4NQO rat model.

It is widely accepted that the TME should be viewed as a vital and active component of cancer progression. The TME can determine the fate of dysfunctional epithelial cells, inducing them to continue to grow, to become normal, or even to be removed^2^. Therefore, strategies aimed at interfering with the cross-talk between tumor cells and their cellular partners in the TME have been considered as potential novel therapies. The greatest advantage of this TME targeting strategy is that non-tumor cells that are relatively genetically stable has the least chance to develop drug resistance^6^. Our results establish an essential role of IL-1β in the TME during oral carcinogenesis. Considering the easy accessibility of oral lesions, the dysplasia stage provides an excellent opportunity to interrupt carcinogenesis. We attempted to confirm whether targeting IL-1β in the TME would effectively prevent oral carcinogenesis. Because cancer chemopreventive agents are mostly intended for high-risk populations, such as oral leukoplakia patients, it is mandatory that these agents have no or minimal side effects. Thus, the commercially available IL-1 receptor antagonist Anakinra, which has been used in the clinic, was employed in our intervention experiment. The IL-1 receptor is expressed in nearly all tissues, and IL-1Ra is an endogenous receptor antagonist that binds to the IL-1 receptor and prevents the binding of IL-1β and IL-1α^8^. In this study, IL-1Ra was injected into the oral submucosa during 4NQO induced oral carcinogenesis in the rat model. Not surprisingly, our results demonstrated that IL-1Ra could attenuate the severity of histopathologic changes in the rat tongue by regulating the expression of key genes to reprogram the TME.

To our knowledge, this is the first study of a TME targeting strategy to this oral carcinogenesis model, and our observations raise several clinical expectations. First, although the effects of IL-1Ra in inhibiting tumor progression have been reported, those conclusions were drawn from data that were collected from *in vitro* or cancer xenograft *in vivo* studies^19^,^20^,^21^,^22^,^23^,^24^,^25^. In the present study, the effect of IL-1Ra was investigated in a carcinogen induced animal model in which malignant transformation is identical to clinical manifestation. We believe that the dynamic variation in all of the carcinogenesis stages of this animal model will provide additional details with clinical implications. Second, the IL-1Ra agent Anakinra, which has been proved useful for rheumatoid arthritis management, has recently been repositioned as an anticancer drug in myeloma and for treatment of Castleman’s disease^26^,^27^. Repositioning IL-1Ra as a cancer preventative or therapeutic agent in OSCC has great advantages in terms of lower drug development costs and risks; more importantly; it can dramatically reduce the time for approval, directly accelerating its application in the clinic to benefit patients. Finally, the administration of IL-1Ra in this study was by direct injection into the tongue submucosa of oral lesions rather than conventional subcutaneous injection. This submucosal injection aimed to simulate the routine treatment procedure adopted in the clinic for the easy recognition and accessibility of oral premalignant and malignant lesions. Moreover, this application has the simultaneous advantages of a maximum concentration of IL-1Ra in oral tissue and minimum side effects to other systems.

**Figure 4:**
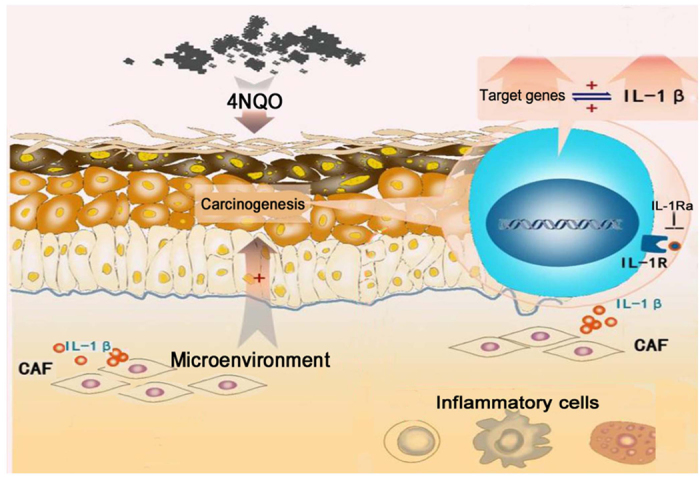
Schematic illustration of the modulation of IL-1β in the tumor microenvironment to promote oral carcinogenesis. Under carcinogenic stimulation, IL-1β is upregulated in both epithelial and stromal compartments such as fibroblasts. Paracrine signals from IL-1β activate gene expression pathways in neighboring cells and vice versa, ultimately setting up a cycle of reinforcement and generating a tumor favorable microenvironment for oral carcinogenesis.

From our results, it is presumed that, under the stimulus of a carcinogen, IL-1β is upregulated in both epithelial and stromal compartments, such as fibroblasts. Paracrine signals from IL-1β activate gene expression pathways in neighboring cells and vice versa, ultimately setting up a cycle of reinforcement and generating a tumor favorable microenvironment for oral carcinogenesis (Fig. 4). Therefore, targeting IL-1β in the TME with IL-1Ra provides a promising chemoprevention strategy to interrupt oral malignant transformation. Although more validation studies must be performed before the application of oral IL-1Ra injection in the clinic, we believe that this novel IL-1Ra medication strategy targeting the TME has practical and translational usefulness in OSCC prevention, and provides a basis for further investigation.

## Materials and Methods

### 4NQO- induced rat oral carcinogenesis model

The 4NQO-induced rat oral carcinogenesis model was established as described previously^28^. Male Sprague-Dawley (SD) rats were divided into two groups. In the experimental group, rats were fed daily with 0.002% 4NQO (Sigma-Aldrich, Saint Louis, MO, USA) solution in their drinking water. At week 14 to week 22, the experimental rats were sacrificed, as visible lesions of tongue dysplasia or SCC had developed, respectively. Rats in the normal control group were fed with distilled water only. The tongues were dissected, and a longitudinal mid-lingual incision was made. Half of the specimens were fixed in 10% buffered formalin, embedded in paraffin and cut into 4-mm sections for hematoxylin and eosin (HE) staining to confirm the pathological diagnosis. The other halves of the specimens were collected for the experiment describe below.

All of the animal procedures were conducted in accordance with the Guidelines for the Care and Use of Laboratory Animals and were approved by the Institutional Animal Care and Use Committee at Sun Yat-sen University.

### Oral epithelia cells and submucosal fibroblasts collection

After subcutaneous tissue and blood were removed, the normal control, dysplasia and SCC lesions were digested in dispase II (1.8 U/mL from Roche, Lewes, UK) at 4 °C overnight. The epithelial layer was enzymatically and mechanically separated from the underlying connective tissue. Next, the epithelial layer was rinsed twice in PBS and stored at −80 °C for subsequent experiments. The underlying connective tissue was cut into small pieces for collagenase digestion (0.1% collagenase type IV, Sigma) at 37 °C for 30 min. After washing twice with PBS, the suspension was finally plated on 6-cm tissue culture dishes in 5 ml of DMEM supplemented with 20% fetal bovine serum. After culturing for 25 min at 37 °C, nonadherent cells were removed to obtain pure fibroblasts. The adherent fibroblasts were immediately collected for subsequent experiments^29^. The purity of the epithelial cells and fibroblasts was confirmed (Supplementary Fig. 6).

### Human tissue sample collection

Sections of paraffin-embedded tissue from routine diagnostic biopsies and operations were used. Specimens were obtained from the oral mucosa of 22 subjects: SCC (n = 7), leukoplasia (n = 5), and healthy control (n = 10). The diagnosis was based on clinical appearance and histology. The study was conducted in accordance with the Declaration of Helsinki. Every subject provided written consent to participate after being informed about the aim and protocol of the research. The study design was approved by the Medical Research Ethical Committees of Guanghua School of Stomatology, Sun Yat-sen University.

### Immunohistochemistry

Immunohistochemical staining was performed according to the manufacturer’s instructions. Briefly, the sections of rat tongue were incubated with a rabbit anti-rat IL-1β polyclonal antibody (1:100, PAB10321; Abnova, CA, USA) and sections of human tissue were incubated with a rabbit anti-human IL-1β polyclonal antibody (1:100, ab2105; Abcam, MA, USA) at 48 °C overnight. Cell and Tissue Staining Kits (R&D Systems, MN, USA) were used to locate and visualize the antigen–antibody complex with diaminobenzidine. Tissue sections were counterstained with Mayer’s hematoxylin. For evaluation of the slides, 100 tumor or epithelial cells were counted per high-power field (original magnification, 200×)^30^. The percentage of positive cells was recorded.

### Cell lines and cell culture

The human tongue SCC cell line Cal27 (American Type Culture Collection, Rockville, MD) was maintained in DMEM high-glucose culture medium (GIBCO, Grand Island, NY, USA) with 10% fetal bovine serum (FBS; GIBCO) at 37 °C in a humidified atmosphere containing 5% CO_2_.

### Establishment of knockdown cell lines

Mission shRNA plasmids for human IL-1β were used with a lentiviral packaging mixture (Forevergen Biosciences, Guangzhou, China). The sequences were shIL-1β as follows: shIL-1β forward, 5′-AACTGGAGAGTGTAGATCCCAAATTCAAGAGATTTGGGATCTACACTCTCCTTTTTTC-3′; reverse, 3′-TTGACCTCTCACATCTAGGGTTTAAGTTCTCTAAACCCTAGATGTGAGAGGAAAAAAGAGCT-5′; scrambled shRNA forward, 5′-AACTTTCTCCGAACGTGTCACGTTTCAAGAGAACGTGACACGTTCGGAGAATTTTTTC-3′; reverse, 5′-TCGAGAAAAAATTCTCCGAACGTGTCACGTTCTCTTGAAACGTGACACGTTCGGAGAAAGTT-3′. Lentiviral-mediated transfection of shRNAs was performed according to the manufacturer’s instructions. Briefly, 293 T cells were co-transfected with the lentiviral vector and packaging vector and the supernatant was collected after 48 and 72 h. After centrifugation for 15 min at 5000 × g, the lentiviruses were recovered and resuspended in PBS before performing the infection with 6 μg/ml of polybrene. Stable cells with IL-1β knockdown were selected following transduction with 2 μg/ml puromycin for 2 weeks. The transfection efficiency of IL-1β knockdown was examined by western blot analysis.

### Protein isolation and western blotting

The details of the protein isolation and western blot analysis have been described in a previous study^31^. A specific anti-human IL-1β antibody (1:1000), an anti-human β-actin antibody (1:1000) from Cell Signaling Technology (Danvers, MA, USA) and horseradish peroxidase conjugated goat secondary antibodies (Beyotime Institute of Biotechnology, Haimen, Jiangshu China) were used in this study.

### Cell viability assay

Cell viability was determined by using an MTT assay (Roche Diagnosis) in 96-well plates (4,000 cells/well) by following the instructions of the manufacturer. Each experiment was performed in triplicate and repeated three times.

### *In vivo* tumor xenograft model in nude mice

Fourteen BALB/c-nude mice, 5–6 weeks old and 20 g in weight, provided by Guangdong Medical Laboratory Animal Center (Guangzhou, China) were maintained in accredited animal facilities at the Animal Laboratory, Zhongshan Ophthalmic Center, Sun Yat-sen University (Guangzhou, China). For the tumorigenic assay, all 14 mice (7 mice/group) underwent subcutaneous injection of 100 μl (3.5 × 10^6^) of viable cell suspension with LV-shIL-1β or LV-shNC in the dorsal scapula region. The size of the tumors was measured by a researcher blind to the identity of the animals every 4days with calipers, and the tumor volume was determined using the simplified formula of a rotational ellipsoid (L × W^2^/2), where L is the largest dimension and W is the perpendicular diameter. Upon termination of the experiment, the mice were sacrificed, and individual tumors were weighed and collected for further analysis.

### IL-1 Ra intervention in the 4NQO- induced rat oral carcinogenesis model

Rats were exposed to 4NQO for 14 weeks and then randomly distributed into 4NQO and IL-1Ra intervention group. After intraperitoneally anesthesization with chloral hydrate, the IL-1Ra intervention group (n = 30) received a tongue submucosal injection of 100 μl of rat recombinant IL-1Ra (R&D Systems, Minneapolis, MN, USA) at a concentration of 3.5 μg/ml in the middle-third of the tongue, while the 4NQO group (n = 15) received an equal volume of the diluent (sterile saline) weekly from week 14 to week 22. The rats in the control group (n = 5) were fed with distilled water only from 0 to 22 weeks. At the end of week 22, the rats were sacrificed and tongues were dissected and a longitudinal mid-lingual incision was made. One half of each tissues sample was prepared for histological study as described previously, while the other half was stored at −80 °C for subsequent experiments. The study was approved by the Animal Use and Care Committees of Guanghua School of Stomatology, Sun Yat-sen University.

### Histopathologic assessment of rat tongue samples

In this study, we followed a procedure that was similar to that in a previous study. The distance between the tip of the tongue to its most posterior aspect was divided into three equal parts, which were designated the ‘anterior’, ‘middle’ and ‘posterior’ thirds. The histopathological changes were classified for each third on an 8-point scale to score changes ranging from normal-looking epithelia (a score of 0), to invasive carcinoma (a score of 7)^30^. The results were expressed as the mean score of the histopathological changes for each ‘third’ for the 4NQO and IL-1Ra intervention groups.

### RNA isolation and purification

The total RNA from oral epithelia, fibroblasts and tongue tissue was extracted with Trizol reagent (Invitrogen, Gaithersburg, MD, USA) and further purified using the Qiagen RNeasy Mini Kit (Qiagen, Hilden, Germany) according to the manufacturer’s instructions. The RNA quality was assessed by formaldehyde agarose gel electrophoresis and was quantitated spectrophotometrically.

### Microarray experiments

The gene expression profile was examined by microarray (Affymetrix Gene Chip Rat Genome 230 2.0 Array, Human Genome U133 Plus 2.0 Array) at Capitalbiochip Corporation (Beijing, China), where the gene chip microarray service is certified by Affymetrix. The protocol for microarray processing was conducted according to the GeneChip Expression Analysis Technical Manual (Affymetrix, 701021, Rev.5). The hybridized microarrays were then scanned using the GeneChip Scanner 3000 and converted into TIFF images in preparation for analysis.

### Data processing and analysis

The microarray data were processed and analyzed as described previously^31^. A differentially expressed gene was defined as a variation in gene expression no less than 2-fold or 1.5-fold with a detection *P* value of less than 0.05. The gene expression data discussed in this publication have been deposited in Gene Expression Omnibus. The bioinformatics analysis including hierarchical clustering, biological process Gene Ontology (GO) analysis, and KEGG pathway analysis were conducted as described previously.

### Biological association network analysis

BANs analysis was conducted using GeneSpring GX software v11.0 (Agilent Technologies, Santa Clara, CA, USA). This software package generates interaction networks, starting with a differentially expressed gene list, by the information present in a database of known molecular interactions. Details of this network construction have been described in a previous study^31^.

### Delineation of the interaction network of differentially expressed genes

Details of the network construction are described in a previous study^32^. Differentially expressed genes were input into Bioinformatics Database STRING (http://string-db.org) to construct the interactions network of proteins encoded by these genes. Subsequently, the network data were input into Cytoscape 2.6.2. The central nodes were exposed and marked and the network was visualized.

### Statistical analysis

Data collection and statistical analysis were performed using SPSS 13.0 statistical software (SPSS Inc, Chicago, IL, USA). All of the data are presented as the mean ± standard deviation (SD) of triplicate determinations. Data were analyzed using Student’s t test and ANOVA when appropriate. A *P* value less than 0.05 was considered to be statistically significant. Each experiment was repeated three times, with similar results.

## Additional Information

**How to cite this article**: Wu, T. *et al.* Modulation of IL-1β reprogrammes the tumor microenvironment to interrupt oral carcinogenesis. *Sci. Rep.* **6**, 20208; doi: 10.1038/srep20208 (2016).

## Supplementary Material

Supplementary Information
