# Supplementary material for: Modulation of IL-1β reprogrammes the tumor microenvironment to interrupt oral carcinogenesis
Source: Sci Rep. 2016 Feb 1;6:20208. doi: 10.1038/srep20208 (PMC4735323; doi:10.1038/srep20208)
Supplement: Supplementary Information [file srep20208-s1.pdf]

Modulation of IL-1β reprogrammes the tumor microenvironment to interrupt oral carcinogenesis

Tong Wu†, Yun Hong†, Lihua Jia, Jie Wu, Juan Xia, Juan Wang, Qinchao Hu, Bin Cheng*

Department of Oral Medicine, Hospital of Stomatology, Sun Yat-sen University, Guangzhou, China.

Guangdong Provincial Key Laboratory of Stomatology, Guanghua School of Stomatology, Sun Yat-sen University, Guangzhou, China.

* Correspondence and request for materials should be addressed to Bin Cheng. Tel: +86 20 83741891; Fax: +86 20 83822807; Email: chengbin@mail.sysu.edu.cn

† These authors contributed equally to this work.

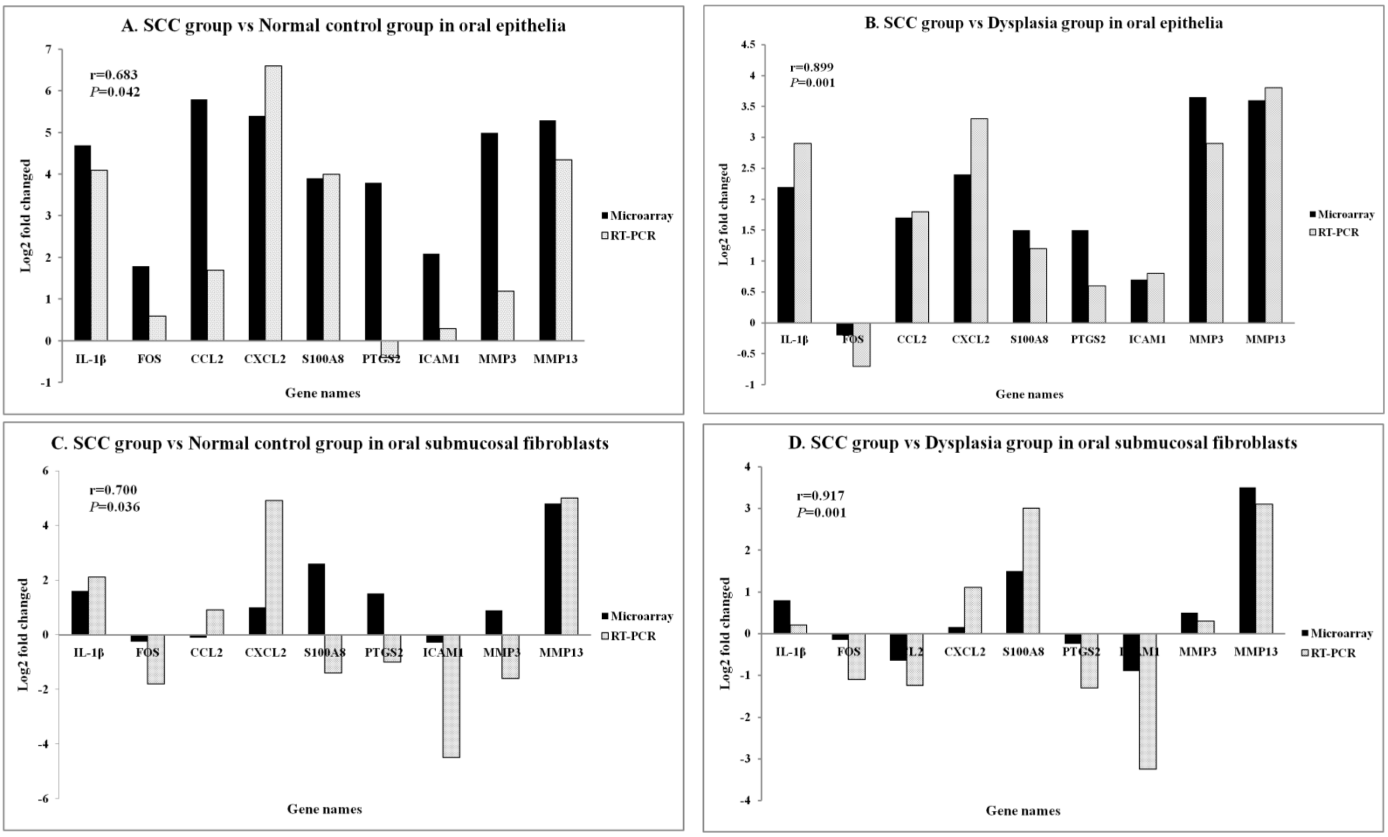

Supplementary Figure 1. Real time PCR validation of selected differentially expressed genes identified by microarray in oral epithelia and fibroblasts during 4NQO induced oral carcinogenesis. A randomly selected subset of 9 genes was used to validate the microarray results. The fold change gene expression measured by RT-PCR was then compared directly to the fold change obtained by Affymetrix hybridization. The Spearman correlation between the two methods was tested; the r and *P* value were shown in figures. (A) SCC group vs Normal control group in oral epithelia. (B) SCC group vs Dysplasia group in oral epithelia. (C) SCC group vs Normal control group in oral submucosal fibroblasts. (D) SCC group vs Dysplasia group in oral submucosal fibroblasts.

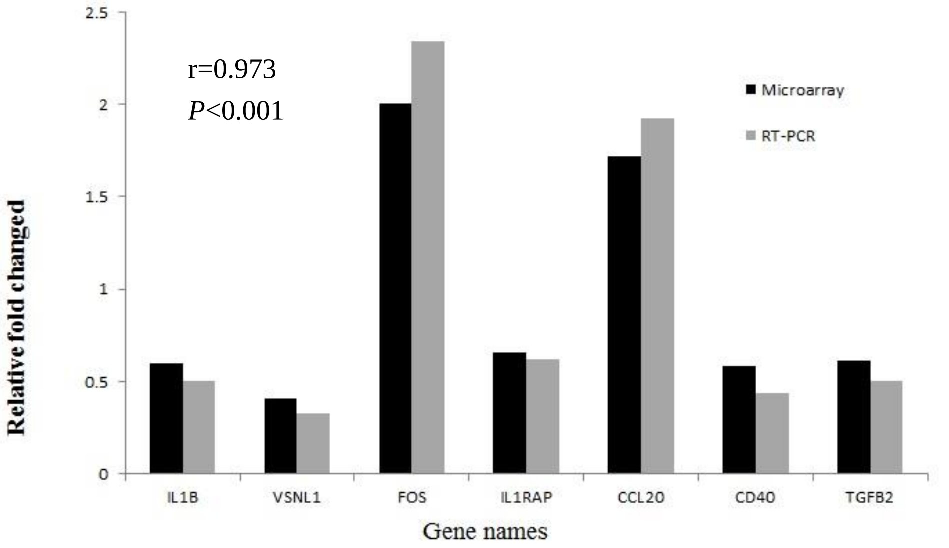

Supplementary Figure 2. Real time PCR validation of selected differentially expressed genes identified by microarray in OSCC Cal27 cells with and without IL-1β shRNAs transfection. A randomly selected subset of 7 genes was used to validate the microarray results. The fold change gene expression measured by RT-PCR was then compared directly to the fold change obtained by Affymetrix hybridization. The Spearman correlation between the two methods was tested; the r and *P* value were shown in figures.

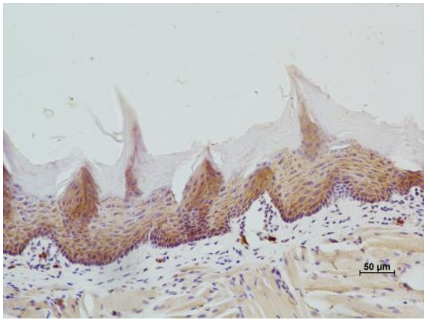

Supplementary Figure 3. IL-1 receptor I (IL-1RI) expression rat oral mucosa detected by immunohistochemical staining (magnification 200×).

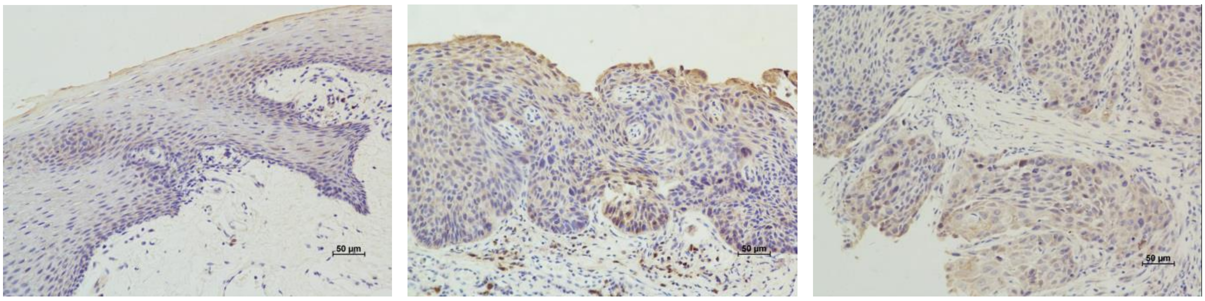

Normal **Dysplasia** **Carcinoma**

Supplementary Figure 4. IL-1 receptor I (IL-1RI) expression during human oral malignant transformation detected by immunohistochemical staining (magnification 200×).

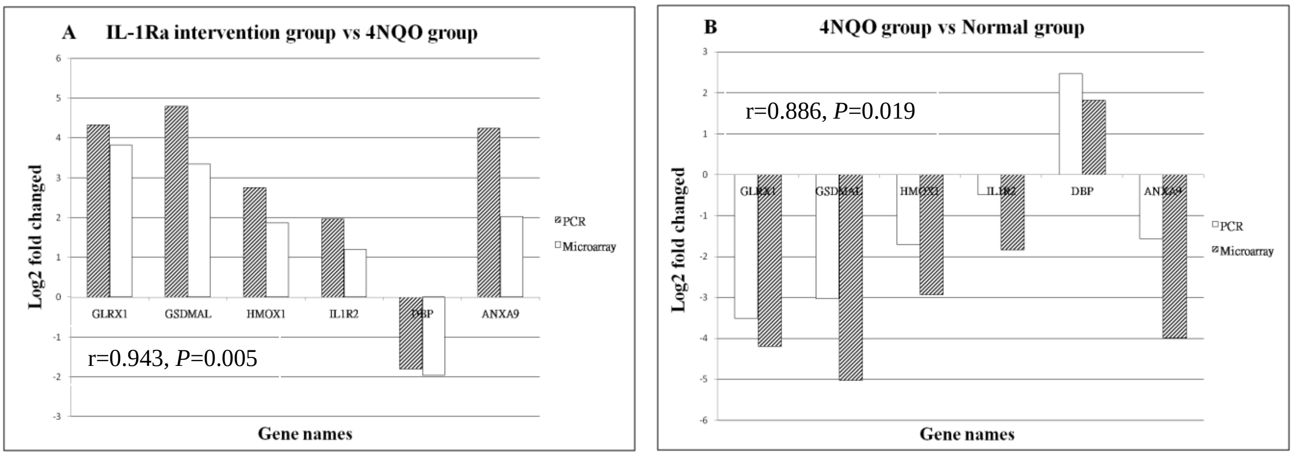

Supplementary Figure 5. Real time PCR validation of selected differentially expressed genes identified by microarray after IL-1Ra administration in 4-NQO induced oral carcinogenesis. A randomly selected subset of 6 genes was used to validate the microarray results. The fold change gene expression measured by RT-PCR was then compared directly to the fold change obtained by Affymetrix hybridization. The Spearman correlation between the two methods was tested; the r and *P* value were shown in figures. (A) IL-1Ra intervention group vs 4NQO group in tongue tissue. (B) 4NQO group vs normal group in tongue tissue.

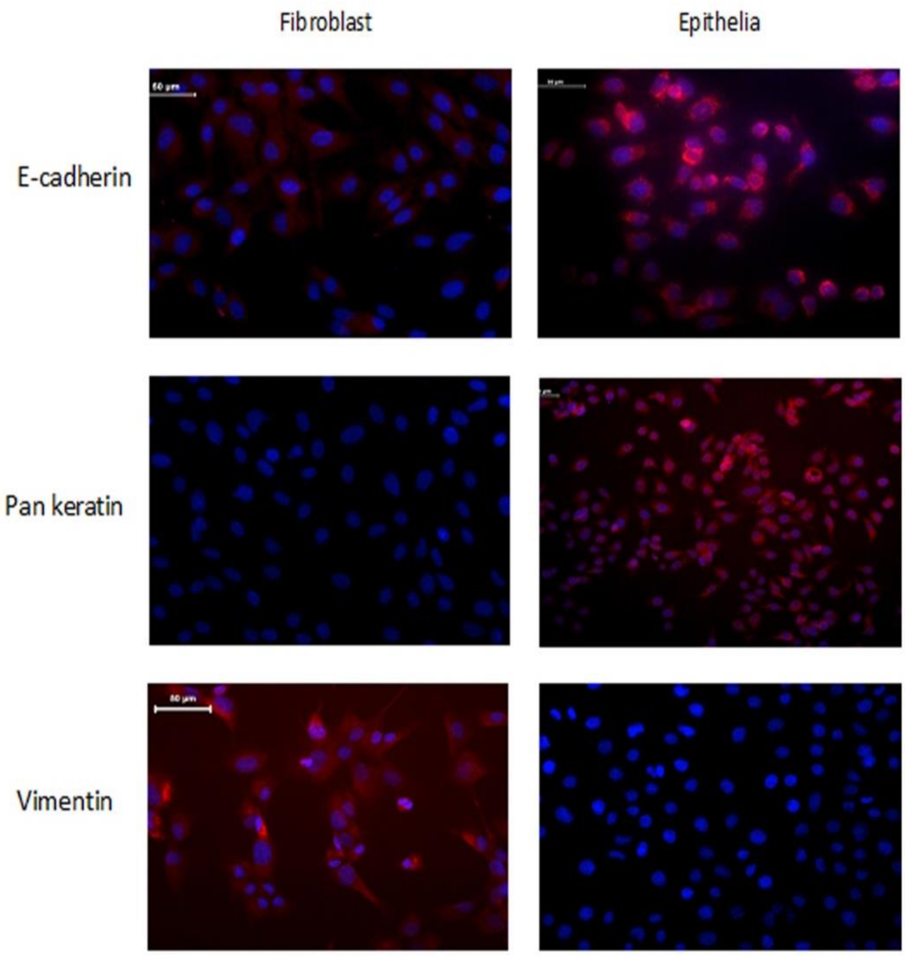

Supplementary Figure 6. Isolation of the epithelial cells and fibroblasts from rat oral mucosa. Representative images of immunocytochemical staining for rat E-cadherin, pan keratin, and vimentin. Blue, DAPI nuclear stain; Red, E-cadherin, pan keratin, and vimentin.

Supplementary Table 1. Primer designed for quantitative realtime polymerase chain reaction (PCR) analysis

Gene	Primer sequence
IL-1B	5′-GCAGCATCTCGACAAGAGCTT-3′
	5′-GCTCCACGGGCAAGACATAG-3′
FOS	5′-TGGAGCCGGTCAAGAACATT-3′
	5′-GCCGGAAACAAGAAGTCA TCA-3′
CCL2	5′-CGCTTCTGGGCCTGTTGTT-3′
	5′-TGGGATCATCTTGCCA GTGA-3′
CXCL2	5′-CGCCCAGACAGAAGT CATAGC-3′
	5′-TTTGGACGATCCTCTGAACCA-3′
ICAM1	5′-CGGGATGGTGAAGTCTGTCA-3′
	5′-GTGCACGTCCCTGGTGATACT-3′
MMP3	5′-CTGGGCTATCCGAGGTCATG-3′
	5′-TTCTGGACGGTTT CAGGGAG-3′
PTGS2	5′-CTACCATCTGGCTTCGGGAG-3′
	5′-ACAGTCGCTCGT CATCCCA-3′
S100a8	5′-GCCTTGAGCA ACGTCATTGA-3′
	5′-TCCTGAAGTCATCCCTGTAGAGG-3′
VSNL1	5′-TGTTTGACGTTTCAGAGGCT-3′
	5′-AGATGTGTGTAAGGGTAGATTTAGT-3′
IL1RAP	5′-GGGTGGTGGCTGCTATTTCT-3′
	5′-AGCTACCTTTGAACCACGGG-3′
CCL20	5′-TCAGAAGCAGCAAGCAACTTTGAC-3′
	5′-TTCGTCGGCCATCTGTGTT-3′
TGFB2	5′-GTATTGCTCTGCAATGGGCG-3′
	5′-CAGCACATGTTTTCTGGGGC-3′
CD40	5′-ATACTGTCTGCCAACCCTGC-3′
	5′-TCCTTTGGTTTCTTGACCACCT-3′
MMP2	5′-CGGTTTATT TGGCGG ACA GT-3′
	5′-GCCTCGTACACGGCATCAAT-3′
GLRX1	5′-GCATAGGCGGATGCAGTGAT-3′
	5′-ACATCAACTCTCAGGGGCAC-3′
GSDMAL	5′-GTGGAAACCGTGCAGGAAGT-3′
	5′-GGGATGTCCCACTCATCTTT-3′
HMOXL	5′-CTCTATCGTGCTCGCATGA-3′
	5′-GGGACTCTGGTCTTTGTGTT-3′
IL1R2	5′-CAAGGATGTGGGTGAAGGAT-3′
	5′-GGAAAGAGGCTTCGGTATTC-3′
DBP	5′-ACCGTGGAGGTGCTAATGAC-3′
	5′-AGGCTTCAATTCCTCCTCTGA-3′
ANXA9	5′-ATGGGTCCTGCTCTTTACACA-3′
	5′-CCGGATAGCATCCTCCAGTT-3′
β-actin	5′-TGCCTCTGGTCGTACCACTG-3′

(control)	5'-GCGAAACCTTCGTAG ATGGG-3'

RNA preparations used for the microarray were also subjected to realtime PCR validation assays using primers specific for a randomly selected cohort of those genes. The primer sets for these genes were designed to amplify about 100 bp segments and primers specific for β-actin were used as a housekeeping gene control. All primers were custom-designed from GenBank mRNA sequences and prepared by DaAn Gene Company (Guangzhou, China). Their sequences are listed above. cDNA was synthesized from 4 mg RNA samples using oligo dT to prime the reverse transcriptase reaction. RT-PCR was carried out on the ABI 9700 instrument (ABI company, Foster City, CA, USA) with the QPCR SYBR green detection reagent (ABI company) for three minutes at 938C for the initial denaturing, followed by 40 cycles of 93℃ for 30 s, 55℃ for 45 s and 72℃ for 45s. Cell threshold values for each gene were determined and fold induction compared with RPS26 was calculated using the ΔΔCT method. RT-PCR results were recorded for each gene as fold change versus control. PCR products were evaluated by dissociation curves to confirm single amplication and the absence of significant primer-dimer contamination.
